# Human cardiac myosin light chain 4 (MYL4) mosaic expression patterns vary by sex

**DOI:** 10.1038/s41598-019-49191-0

**Published:** 2019-09-03

**Authors:** Tony Y. Wang, Dan E. Arking, Joseph J. Maleszewski, Karen Fox-Talbot, Tim O. Nieuwenhuis, Lakshmi Santhanam, Renu Virmani, Avi Z. Rosenberg, Marc K. Halushka

**Affiliations:** 1 Division of Cardiovascular Pathology, Department of Pathology, Johns Hopkins University School of Medicine, Baltimore, MD USA; 2 McKusick-Nathans Institute of Genetic Medicine, Johns Hopkins University School of Medicine, Baltimore, MD USA; 3 Department of Laboratory Medicine and Pathology, Mayo Clinic, Rochester, Minnesota USA; 4 Department of Anesthesiology and Critical Care Medicine, Johns Hopkins University School of Medicine, Baltimore, MD USA; 5 CVPath Institute, Gaithersburg, Maryland USA; 6 Division of Renal Pathology, Department of Pathology, Johns Hopkins University School of Medicine, Baltimore, MD USA

**Keywords:** Cardiovascular biology, Proteomic analysis

## Abstract

Sex disparities modulate cardiac function, although the proteins and mechanisms remain to be elucidated. We recently demonstrated a mosaic pattern of protein expression in the heart for over 100 proteins. Here we investigate one of these proteins, myosin light chain 4 (MYL4), which is important for contractile functions by increasing force production. We assayed the expression pattern of MYL4 across 756 ventricular myocardial samples from 668 individuals utilizing a semi-automated Cell Profiler method on five tissue microarrays (TMAs) of cardiac tissues across a diverse set of diseases. The percentage of MYL4 positive cells was significantly higher in male subjects independently across all five TMAs, regardless of disease state (p = 8.66e-15). Higher MYL4 expression was also modestly associated with hypertrophic cardiomyopathy (p = 6.3e-04). MYL4 expression did not associate with sudden cardiac death or other cardiomyopathies. This study demonstrates a new mosaic pattern of protein expression that underlies sex disparities in the human heart.

## Introduction

Sex disparities in cardiac function have long been recognized. These have been identified at the level of Ca^2+^ handling with differences in protein levels, contraction times, relaxation times, and myofilament responsiveness^1–3^. Myocyte cell volume also differs between men and women with cell hypertrophy occurring at different rates along with concomitant apoptosis of myocytes^4^. Sex hormones have been implicated in regulating cardiac natriuretic hormones and modulating cardiac fibrosis^5,6^. The summation of these effects are disparities in clinical outcomes between men and women including higher rates of female mortality in congestive heart failure^7^.

The relationship between sex and contractile proteins, such as myosins, is less established. A study of rats demonstrated higher levels of myosin heavy chain 7 (*MYH7*) among male rats with left ventricular hypertrophy (LVH) compared to female rats with LVH^8^. In a second study of gene expression differences by sex in human dilated cardiomyopathy, *MYH7* was 2.5 fold increased in women and myosin light chain 4 (MYL4) was 1.7 fold decreased^9^. *MYL4* (ALC1, MLC1) has historically been classified as a myosin of the atria and embryonic heart. It has an important role in the contractile mechanisms of the sarcomere by increasing cross-bridge kinetics and increasing sensitivity to Ca2+, allowing for greater force production^10^.

We recently demonstrated an interesting mosaic protein expression pattern of MYL4^11^. Consistent with established literature, MYL4 expression is robust and homogeneous in the atria. Additionally, it is universally present in fetal and neonatal ventricular tissues. However, from infancy until roughly 5 years of age, the percent of ventricular cardiomyocytes expressing MYL4 decreases from 100% to roughly 15%, causing a mosaic pattern of expression. This pattern had been seen in a limited scale elsewhere^12^. However, the functional significance of these patterns is unknown^11^.

Despite the general localization of MYL4 to the atria, MYL4 has been described in ventricular tissues as being involved in structural ventricular changes and disease^13^. It has been proposed that MYL4 upregulation is more accurately described as an isomeric switch from myosin light chain 6 (MYL6), also known as ventricular light chain-1 (VLC-1)^10^. *MYL4* expression is significantly increased in patients with hypertrophic cardiomyopathy (HCM)^14^, ischemic heart disease (IHD)^15^, and dilated cardiomyopathy (DCM)^15^. In patients with aortic stenosis or aortic insufficiency, *MYL4* expression is increased, and expression is subsequently decreased following aortic valve replacement^16^. Increased MYL4 expression has even been found in congenital heart diseases such as Tetralogy of Fallot (TOF), double outlet right ventricle (DORV), and infundibular pulmonary stenosis (IPS)^17^. Thus, upregulation may be a general marker of cardiac stress or a structural change with a need to improve contractile function.

Functional studies of cardiac muscle fibers show increased MYL4 expression is associated with increased contractility. Fibers sampled from patients with congenital heart defects TOF, DORV, and IPS had higher maximal velocity and an increased rate of shortening^17^. Additionally, fibers from failing hearts with DCM or IHC showed a positive correlation between MYL4 concentration and Ca^2+^ responsiveness. Interestingly, in that study, the amount of MYL4, as a percent of total myosin light chains, varied between 0 and 10%^15^. These studies suggest the importance of MYL4 to disease or phenotypic differences between individuals but fail to appreciate sex disparities in MYL4 expression.

If only a subset of ventricular cells express MYL4, how do global changes in MYL4 expression, described above, occur? Rather than global upregulation, it may be that more cells activate the MYL4 gene resulting in increased MYL4 positive cells noted by immunohistochemistry. To investigate this, we evaluated five cardiac tissue microarrays (TMAs) comprising cases of sudden cardiac death (SCD), HCM, arrhythmogenic cardiomyopathy (ACM), IHD, DCM and control tissues from men and women. We hypothesized the percent of MYL4 positive cells may vary between disease states or other phenotypes. To perform this analysis, we generated a new semi-automated CellProfiler tool to establish the percentage of MYL4 positive myocytes in each tissue.

## Results

### Patient demographics

In total, 787 heart tissue cores were present on five TMAs. Due to some unevaluable images (tissue folds, loss, etc.), 756 samples from 668 individuals were evaluated. As seen in Table 1, 292 control subjects and 494 non-control subjects across 9 disease categories comprised the five TMAs. SCD cases (311) were segmented based on coronary artery disease status and the presence/absence of thrombosis at death. Overall, the cohort was 69.2% male and 60.8% Caucasian. Most subjects were between 40–75 years of age.

**Table 1:** Patient demographics of heart samples across 5 TMAs.

Disease	Individuals	Heart Cores Evaluated	% Male	% Caucasian	Age (±SD)
Control	245	284	61.3%	76.5%	54.7 ± 16.4
SCD + CAD - Thrombosis	118	133	85.7%	69.6%	55.8 ± 10.8
SCD - CAD	85	91	68.1%	57.5%	48.4 ± 13.6
SCD + CAD + Thrombosis	70	83	85.5%	68.8%	48.0 ± 10.1
HCM	55	59	52.5%	69.5%	54.9 ± 13.7
DCM	41	45	64.4%	42.2%	55.9 ± 13.8
IHD	34	35	71.4%	85.7%	61.0 ± 9.1
ACM	9	14	71.4%	72.7%	42.6 ± 12.4
Pediatric Congenital	8	8	50.0%	50.0%	3.9 ± 3.4
Other^a^	14	15	53.3%	35.7%	51.8 ± 12.5

Key. SCD = Sudden Cardiac Death; CAD = Coronary Artery Disease; CM = Cardiomyopathy; ^a^includes cardiomegaly, chronic active myocarditis, right heart failure, noncompaction cardiomyopathy, retransplant, sarcoidosis, and other unclassified disease.

### Validation of CellProfiler analysis and heterogeneity in sample staining

The CellProfiler system determined the percentage of all pigmented pixels to total pixels, but not specifically the number of brown cells. To determine the correlation of these measures, we compared blinded manual counting of MYL4 positive cells to total cells in seven cores. We compared ImageJ Cell Counter % MYL4+ cells, with CellProfiler % pigmented area scores. The correlation between the two methods was r = 0.94, indicating that CellProfiler accurately determined the percent positive cells and could be used to evaluate all cores (Supplementary Fig. S1).

To ensure reproducibility in our methodology of scoring percentage mosaicism using only small representative heart cores, we measured, in a blinded fashion, the MYL4 percentage mosaicism of two (N = 78) or three (N = 4) separate ventricular heart cores taken from the same heart across 82 patients. Five duplicate cores on the All Cardiomyopathy TMA (TMA4) were septal/left ventricular comparisons. The average variation in percent MYL4+ staining between replicate cores was 3.5% (Supplementary Fig. S2).

### A diverse range of mosaicism in MYL4 staining

**Figure 1:**
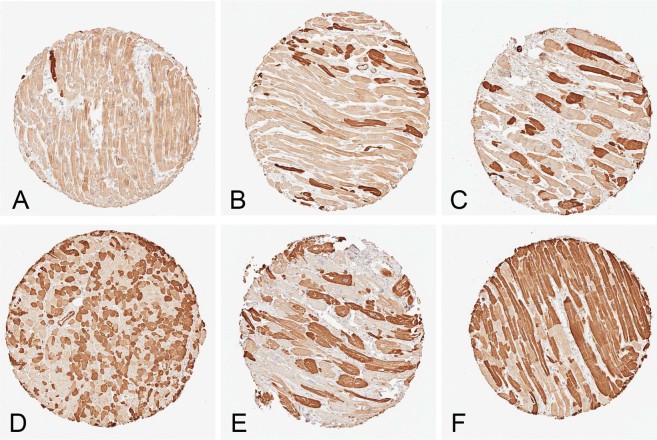
Representative images of mosaic MYL4 staining patterns in various cardiac diseases. (**A**) Female, sudden cardiac death, 1% MYL4+ (**B**) Female, non-cardiac cause of death, 13.8% MYL4+ (**C**) Male, ACM, 32.3% MYL4+ (**D**) Male, non-cardiac cause of death 48.2% MYL4+ (**E**) Male, hypertrophic cardiomyopathy, 55.9% MYL4+ (**F**) Male, non-cardiac cause of death 78.7% MYL4+. All images taken from the same TMA.

We binarized MYL4 expression in cardiomyocytes between ‘off’ (no to blush staining on IHC) and ‘on’ (moderate to strong intensity staining). Adult hearts had a wide range of mosaicism (Fig. 1). MYL4 expression across the cohort ranged from a minimum of <1% of cells to a maximum of 92% of cells positive for MYL4. The average of the cohort was 14.7%. Across the 610 left ventricle cores, the average percent of the MYL4+ cells was 14.6%. For the 81 septal tissues, the average value was 16.2%.

### Male subjects have higher levels of MYL4

**Figure 2:**
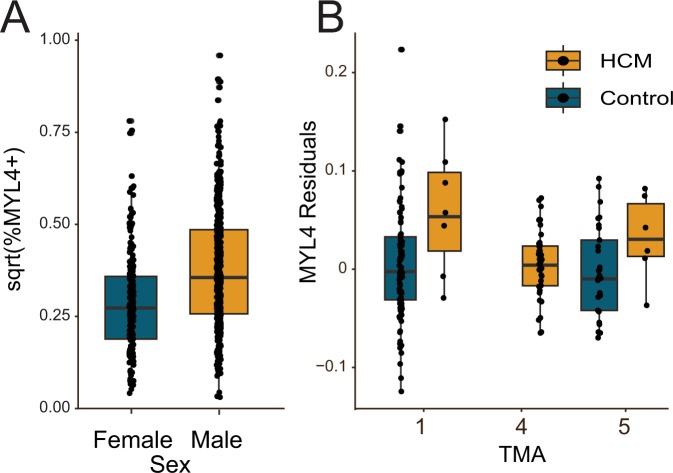
MYL4+mosaicism by gender and disease. (**A**) Male subjects had more MYL4+ cells, independent of disease state (p = 8.66e-15). (**B)** In left ventricular segments, MYL4+ cells were increased in HCM compared to control tissues (TMAs 1&5). There were no control septal tissues to compare to the HCM cases on TMA4. The MYL4 residual is of a regression of sqrt(%MYL4+) cells adjusted for multiple measures per individual and effects of different TMAs, age, and sex.

Across all five TMAs regardless of disease or control groups, male subjects had an increased percentage of MYL4+ cells, despite a wide diversity in MYL4 positive cells (p = 8.66e-15, linear mixed model accounting for sex and age, Table 2). (Fig. 2A and Supplementary Fig. S3). A linear mixed model that incorporated heart weight (which was missing for 308 subjects), showed that heart weight was also associated with MYL4 positivity (p = 4.52e-03) (Supplementary Fig. S3), but had little effect on sex (p = 2.13e-14).

**Table 2:** Linear mixed model for all 756 samples across five TMAs.

	Estimate	Std. Error	t. value	p.z
(Intercept)	0.29	0.025	11.539	0
Age	−0.00013	0.00041	−0.31	0.76
Sex	0.097	0.013	7.76	8.66e-15

There is a strong correlation between patient sex and the percent of MYL4 positive cells.

### Hypertrophic cardiomyopathy subjects have increased left ventricular MYL4+ cells

In seven subjects in TMA1 we noted an increase in MYL4 positivity in HCM relative to control subjects (Fig. 2B). This was affirmed in a linear mixed model with covariates of age, sex, SCD status, ACM and HCM, (p = 9.6e-05 for HCM, Table 2). We tried to replicate this using just septal tissues, where we would have increased number of samples as the result of the more common septal myectomies for HCM. However, we did not have a ready source of control septal tissues, thus HCM was compared only to other cardiomyopathies on TMA4. In this second comparison, the percent of MYL4+ cells in septal tissue of HCM (N = 45) was not different in comparison to other forms of cardiomyopathy (p = 0.6, linear mixed model with age, sex and HCM) (Supplementary Fig. S3). We therefore attempted to replicate the TMA1 data on a second left ventricular-based TMA and obtained an additional eight HCM subjects and compared these samples to new controls on TMA5. Here we replicated the significant increase in MYL4+ staining in HCM (Fig. 2B). Across the entire dataset, including all control and HCM samples from TMAs 1, 4 and 5, HCM was significantly positive (p = 6.3e-04, linear mixed model including age and sex covariates).

### No correlation of MYL4 expressivity with adult age or ethnicity

We compared the percent of MYL4+ cells with patient age in the adult population. We observed no correlation between percent positivity and patient age (p = 0.76, linear mixed model with sex as a covariate) (Supplementary Fig. S3). Additionally, within our population we compared the percent of MYL4+ cells between Caucasian and African American subjects, finding no statistical difference p = 0.63.

### No strong correlation of MYL4 and SCD, ACM, DCM and IHD

We compared the percent of MYL4 positive cells across all SCD (N = 317) and controls (N = 219) across TMAs 1 & 2. There was no overall significant difference in MYL4+ cells (Supplementary Fig. S3). In a linear mixed model, there was a weak signal for reduced percentage of MYL4+ cells in individuals dying of SCD with no coronary artery disease (p = 0.012, Table 3). There was no significant finding for IHD (p = 0.19) and DCM (p = 0.66) in a model with age, sex and HCM (Supplementary Table S1).

**Table 3:** Linear mixed model evaluating TMAs 1 & 2 for correlation with MYL4 positivity.

	Estimate	Std. Error	t. value	p.z
(Intercept)	0.31	0.27	11.47	0
Age	−0.00037	0.00044	−0.84	0.40
Sex	0.090	0.015	5.94	2.93e-09
SCD + CAD - Thrombosis	0.011	0.018	0.63	0.53
SCD - CAD	−0.049	0.019	−2.52	0.012
SCD + CAD + Thrombosis	−0.031	0.021	−1.46	0.15
HCM	0.21	0.054	3.90	9.6e-05
ACM	−0.028	0.045	−0.62	0.53

Male sex and HCM were significantly associated with MYL4 positivity along with a weaker signal for SCD without CAD.

### The MYL4 regulatory environment does not explain variable myocyte expression

Based on the strong sex-based differences, we explored the potential role of hormones as regulators of MYL4. We identified transcription factor binding sites in the enhancer regions of MYL4 and cross referenced this list with known sex hormone-related transcription factors identifying NR2C2 (TAK1, TR4), NR4A1 and NR2F2 (COUP-TFII) as the most frequently noted TFs in the locus^18^. NR2C2 and NR2F2 are both female-biased transcription factors, with NR2C2 acting as a repressor of thyroid hormone and estrogen receptor pathways^19,20^. Previously, a knockout of NR2F2 was shown to cause ventricularization of the atria^21^. We hypothesized that MYL4+ ventricular cells, under the control of NR2F2 may have a more atrial phenotype. Previously the atrial phenotype was described as smaller myocytes with fewer t-tubules. We scored the area of 110 MYL4+ cells and 231 MYL4- cells from 13 subjects, but found no differences in size in the entire population or by gender (p > 0.05), suggesting NR2F2 was not a key regulator of MYL4 in the ventricles.

## Discussion

For the first time, we demonstrate an association of cardiac mosaicism across hundreds of heart samples to sex. The percent of MYL4+ cells was significantly increased in male hearts across all five TMAs (p = 8.66e-15), independent of disease type, implying strong sex differences in MYL4 expression. This data indicates that a novel regulatory method of activating a protein in only a subset of similar cell types causes functional differences by sex. This adds diversity to mechanisms of sex-related cardiac phenotypes^1,2,22^.

Our data is consistent with old gene array experiments studying sex-related effects on cardiac function. In a study of differential expression of cardiac genes in 102 DCM patients, MYL4 was one of >1,800 genes showing sexual dimorphism. It was reported as 2.5 fold higher in men (adjusted p = 0.015)^22^. A second, smaller expression array study showed a 1.7 fold decrease in MYL4 in females with DCM^9^.

As provocative as this finding may be, we are challenged to identify a mechanism that would cause these gender differences on a cell-by-cell basis. An analysis of MYL4 enhancer elements and transcription factor binding sites failed to undercover an obvious hormonal mechanism. MYL4+ cells are also not altered in size relative to adjacent non-MYL4+ cells. Additionally, as we showed previously, MYL3 is uniformly expressed in ventricular cardiomyocytes and does not vary in the presence of MYL4 expression^11^.

The relationship between MYL4 and disease is much more tenuous. We found no role for MYL4 in SCD (other than SCD without CAD), ACM, DCM or IHD. We identified a marked increase in MYL4 positive cells in HCM only on left ventricular free wall samples using two independent TMAs. However, this was based on only 15 HCM samples. The septal TMA, where we had considerably more cases, failed to demonstrate a notable increase relative to other cardiomyopathy cases. We previously demonstrated MYL4 as having some regional expression differences in the heart, but the role this may play between septum and left ventricle is unknown and major differences are not strongly supported by our TMA data (Supplementary Fig. S2)^11^. Although it is a potentially exciting interaction of increased MYL4 and HCM, validating this will require many more samples than to which we had access.

There are some important limitations and challenges to this work. We were unable to discern the functional significance of MYL4 mosaicism. This is partly due to our inability to perform single cell myocyte experiments in human myocytes in a way in which the MYL4 status of the cell could be known. MYL4 mosaic experiments also could not be performed in mice as we found aged mice (20 months) have uniform MYL4 expression in the ventricles, which contrasts with MYL3 mosaic expression in the atria (data not shown). However, it is known from rodent hearts that single ventricular myocytes can react variably to α1-adrenergic stimulation consistent with a mosaic function^23^.

A final concern about the mosaic pattern is that instead of true mosaicism, it could represent alternative splicing of MYL4 in which the antibody only recognizes one form. Indeed, MYL4 has two transcript variants with alternative 5′ ends. However, the used antibody targets both isoforms. Finally, correlation is not causation and we do not know if MYL4 mosaicism differences by sex or disease status are causative or responsive to other cardiac factors.

## Conclusion

The discovery of one or more clinically relevant correlations for MYL4 positivity opens up a new avenue of functionally important gene regulation in the heart. MYL4 mosaic expression represents an unusual sex-based difference in heart protein expression.

## Methods

### Two sudden cardiac death TMAs (TMAs 1 & 2)

We obtained formalin fixed paraffin embedded human cardiac tissues under approved Investigational Review Board (IRB) protocols from The Johns Hopkins Hospital, CVPath, Inc. and the Mayo Clinic. Tissues were taken from autopsied patients and cardiac surgical specimens including explanted hearts from orthotopic transplantation. All methods were carried out in accordance with relevant guidelines and regulations. Specifically, IRB procedures were followed and the waiver of informed consent was approved for these studies. Two tissue microarrays (TMAs) were generated from left ventricular tissue of autopsied subjects. Autopsied tissues were taken from SCD subjects and matched non-cardiac death controls. Non-cardiac deaths included cancers and accidental deaths. TMA1 was generated with 0.6 mm cores and TMA2 was generated from 1.5 mm cores. TMA1 had additional HCM tissues, ACM tissues and a set of autolyzed tissues from different time points after a transplant and a rapid autopsy^24^. For each heart sample collected, various demographic and clinical data were drawn from the patient chart, including sex, age, ethnicity, diagnosis, heart weight, and BMI, as available.

### ACM TMA (TMA3)

TMA3 was generated of control and ACM tissues. This TMA was made with 1.5 mm cores and contained 63 control tissues and 11 ACM tissues (3 pediatric). This TMA was a combination of autopsy and explanted heart tissues including 30 control tissues replicated from TMA1.

### All Cardiomyopathy septal TMA (TMA4)

TMA4 focused exclusively on ventricular septal tissue was generated. It included 45 HCM, 11 IHD, 17 DCM, and 21 other cases taken from explanted hearts and septal myectomies. For five subjects, matched tissues were taken from the ventricular septum and left ventricular free wall. All tissue cores were 1.5 mm in diameter. For each heart sample collected, various demographic and clinical data were drawn from the patient chart, including sex, age, ethnicity, diagnosis, heart weight, and BMI, as available.

### Cardiomyopathy and control TMA (TMA5)

TMA5 was generated from left ventricular tissues composed of 95 tissues including 33 control heart tissues, 27 DCM tissues, 8 HCM tissues, 24 IHD tissues, 2 sarcoidosis tissues and 1 cardiomegaly tissue. All tissue cores were 1.5 mm in diameter and demographic data of sex, age, ethnicity and heart weight were obtained for each specimen.

### Diagnostic criteria for each disease classification

All determinations of control or disease states of the specimens were made by three cardiovascular pathologists (R.V., J.J.M., and M.K.H). SCD was determined based on the patient history and complete autopsy findings. The evaluation of samples for SCD was described in depth in supplemental material elsewhere^25^, but briefly, CAD was based on at least one coronary artery with >75% luminal narrowing. Thrombus was based on the gross and/or histologic appearance of thrombus and plaque rupture. HCM was based on the gross (thick septum) and microscopic (myocyte disarray, vascular dysplasia, endocardial thickening) findings of explanted hearts. For septal myectomies, HCM was based on the clinical and radiographic findings. DCM was based on clinical and gross cardiac findings. IHD was based on the presence of myocardial scarring consistent with prior myocardial infarction and the presence of CAD. ACM was based on clinical, EKG and gross findings. No diagnoses relied on genetic mutation information, which was not determined for most subjects.

### Immunohistochemistry for MYL4

Heart cores from the four TMAs underwent immunohistochemistry (IHC) for MYL4 as described^11^. Briefly, after paraffin removal, high-temperature antigen retrieval was performed (Trilogy, Sigma Aldrich, St. Louis, MO). Slides were incubated with primary antibodies to Myosin, light chain 4 (MYL4; 1:500; HPA051884; Atlas Antibodies, Stockholm, Sweden) followed by an incubation with a polymer HRP IgG (Leica Biosystems, Pleasanton, CA). The antibody complex was detected with ImmPact DAB (Vector Laboratories, Burlingame, CA) and the slides were counterstained with Hematoxylin (Richard-Allen Scientific, Kalamazoo, MI).

### Calculating percentage of mosaicism with CellProfiler

Stained slides were digitized using an Aperio ScanScope system and were segmented for individual TMA cores. Cells stained variable shades of brown corresponding to high or absent levels of MYL4 construing onto the tissue a “mosaic tile” or “mosaic” pattern. To quantify the extent to which each heart tissue expressed this dimorphism, we generated a new IHC quantification method in CellProfiler^26^. Using CellProfiler, we calculated the “percentage mosaicism” as the ratio of the area of myocytes overexpressing MYL4 (dark brown) to the total area of all myocytes (all shades of brown), (Supplementary Fig. S1). First, we reviewed all images and manually erased large regions of non-cardiomyocytes present (blood vessels, adipose, fibrosis etc.). Then we set the CellProfiler to have a minimum size threshold of 50 pixels to exclude debris and staining noise. Finally, we generated 11 masks of brown intensity using the global 2-class Otsu thresholding method at intensity thresholds from 1.5 to 2.75 to select for MYL4+ cells and one additional mask at intensity threshold 0.5 to select for all myocytes. Because of variable baseling brown intensity and tissue disturbances from core to core, we manually determined which threshold best approximated the area of the MYL4+ myocytes in a blinded fashion.

### Manual counting of pigmented cells

Seven cores were selected from the overall set of TMA images that represented a wide range of MYL4 mosaic expression. The ImageJ Cell Counter plug in was used to count every pigmented and non-pigmented cell in a TMA core by two blinded investigators^27^.

### Statistical analyses

All analyses were performed in R version 3.4.4. To account for multiple spots on the TMA per individual, we used linear mixed models (R package ‘lme4’) with individual ID and TMA number as random effects. Fixed effects included age, sex, and disease status. Heart weight and BMI were log transformed and percent staining was square root transformed (sqrt(%MYL4+) to more closely approximate a normal distribution. Subjects <18 years of age (N = 8) were removed from non-technical analyses. Samples missing sex or age data or having unevaluable images (N = 23) were removed from full analysis. Regression analyses were performed generating residuals.

### Calculating cell area

A total of 341 cells were measured for area using ImageJ^27^. This was performed across 13 different core images. The area of MYL4+ cells was compared to the area of MYL4- cells from the same core images using a paired t-test.

### Regulatory transcription factors

Regulatory/transcription factors at the MYL4 promoter/enhancer locus were assessed using GeneHancer^28^. Maximally 219 transcription factors were predicted to have regional binding sites. Transcription factors were cross-referencing with sex hormone-related nuclear receptors.

## Supplementary information

Supplementary Dataset 1
