# Supplementary material for: Human cardiac myosin light chain 4 (MYL4) mosaic expression patterns vary by sex
Source: Sci Rep. 2019 Sep 3;9:12681. doi: 10.1038/s41598-019-49191-0 (PMC6722118; doi:10.1038/s41598-019-49191-0)
Supplement: Supplementary file 1 — Supplementary Dataset 1 [file 41598_2019_49191_MOESM1_ESM.pdf]

Supporting Information

Human cardiac myosin light chain 4 (MYL4) mosaic expression patterns vary by sex

Tony Y. Wang[1], Dan E. Arking[2], Joseph J. Maleszewski[3], Karen Fox-Talbot[1], Tim O. Nieuwenhuis[1,2], Lakshmi Santhanam[4], Renu Virmani[5], Avi Z. Rosenberg, Marc K. Halushka[1]*

1 Division of Cardiovascular Pathology, Department of Pathology, Johns Hopkins University SOM, Baltimore, MD, USA
2 McKusick-Nathans Institute of Genetic Medicine, Johns Hopkins University School of Medicine, Baltimore, MD, USA.
3 Department of Laboratory Medicine and Pathology, Mayo Clinic, Rochester, Minnesota, USA.
4 Department of Anesthesiology and Critical Care Medicine, Johns Hopkins University School of Medicine, Baltimore, MD, USA.
5 CVPath Institute, Gaithersburg, Maryland, USA.

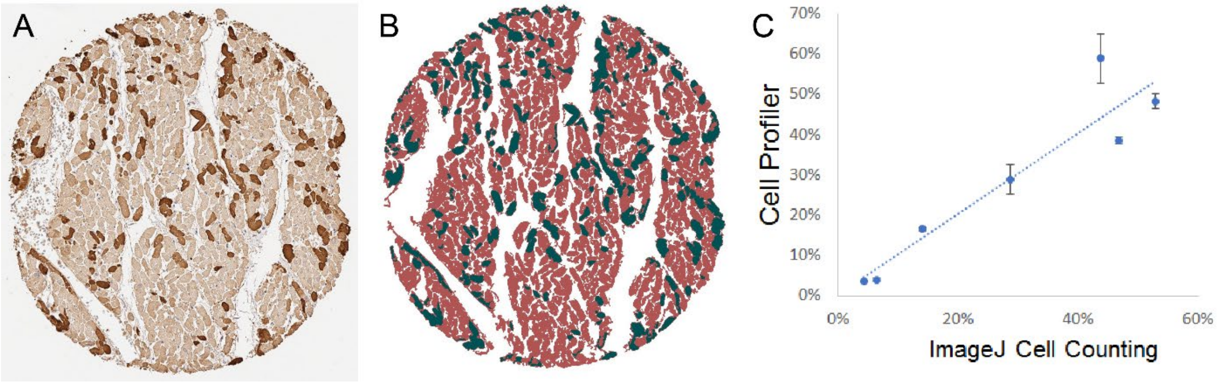

Figure S1: Myocyte categorization using CellProfiler and ImageJ. A) A typical cardiac core showing MYL4+ and MYL4- cells. B) A CellProfiler mask was generated to identify all cardiomyocytes (brown) and positive staining myocytes (green) at different intensity thresholds. An optimal threshold was selected in an operator-dependent fashion. C) A correlation of semi-automated CellProfiler and manual ImageJ Cell Counting methods accurately correlated (r=0.94) with each other on the percent of MYL4+ cells to percent of brown pigmented area.

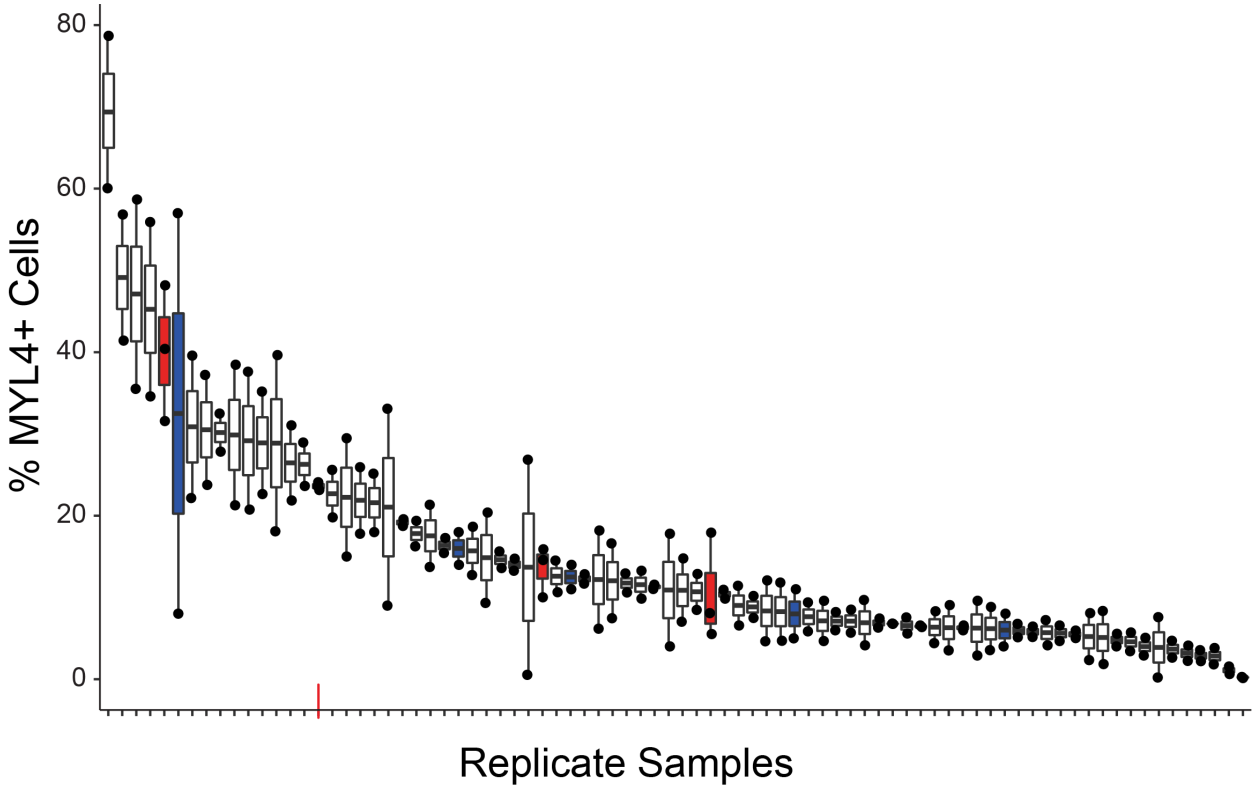

Figure S2. Reproducibility of MYL4 percent positivity by subject. Blue bars are septal – left ventricular comparisons and red bars are triplicate samples. The red line on the x axis indicates one of the triplicate samples.

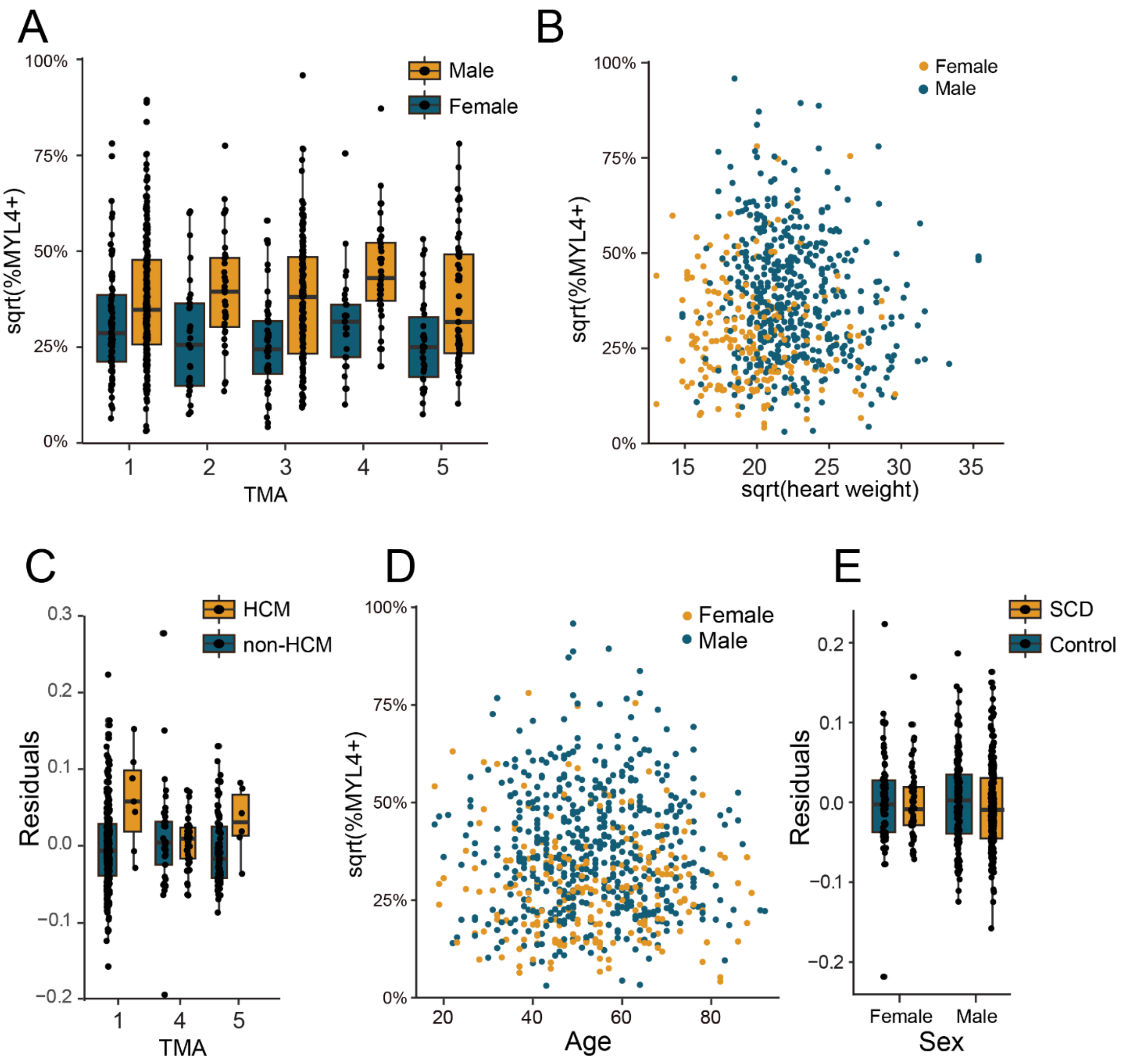

Figure S3. MYL4 correlations by sex, heart weight, HCM status, age and SCD status. A) In each TMA, male MYL4 positivity was increased independently. **B)** There was a significant increase in MYL4 positivity with heart weight (p= 4.52e-03). **C)** Across all samples, HCM MYL4 positivity was increased over all non-HCM samples, but not significantly for septal tissues on TMA 4. **D)** The percent of MYL4+ cells was not related to subject age, when age >20 years. **E)** There was no overall correlation between MYL4 positivity and SCD in men or women.

	Estimate	Std. Error	t. value	p.z
(Intercept)	0.30	0.21	14.28	0
Age	-0.00045	0.00036	-1.23	0.29
Sex	0.096	0.013	7.69	1.51e-14
HCM	0.074	0.021	3.49	4.85e-04
IHD	0.036	0.027	1.31	0.19
DCM	0.011	0.025	0.44	0.66

Table S1. Linear mixed model evaluating TMAs 1-5 for age, sex, HCM, IHD and DCM.
